# Towards understanding human–environment feedback loops: the Atacama Desert case

**DOI:** 10.1098/rstb.2022.0253

**Published:** 2024-01-01

**Authors:** Eugenia M. Gayo, Mauricio Lima, Andone Gurruchaga, Sergio A. Estay, Calogero M. Santoro, Claudio Latorre, Virginia McRostie

**Affiliations:** ^1^ Departamento de Geografía, Universidad de Chile, Santiago 8331051, Chile; ^2^ Center of Applied Ecology and Sustainability (CAPES), Santiago 8331150, Chile; ^3^ Center for Climate and Resilience Research (CR)2, Santiago 8370449, Chile; ^4^ Institute of Ecology and Biodiversity (IEB), Santiago 7750000, Chile; ^5^ Departamento de Ecología, Pontificia Universidad Católica de Chile, Santiago 8331150, Chile; ^6^ Escuela de Antropología, Pontificia Universidad Católica de Chile, Santiago 7821093, Chile; ^7^ Instituto de Ciencias Ambientales y Evolutivas, Universidad Austral de Chile, Valdivia 5090000, Chile; ^8^ Instituto de Alta Investigación, Universidad de Tarapacá, Arica 1001236, Chile; ^9^ Centro PUC Desierto de Atacama (CDA), Santiago 7821093, Chile

**Keywords:** anthroecology, population dynamic theory, ecosystem engineering, social upscaling, boom-and-bust cycles, warfare

## Abstract

The overall trajectory for the human–environment interaction has been punctuated by demographic boom-and-bust cycles, phases of growth/overshooting as well as of expansion/contraction in productivity. Although this pattern has been explained in terms of an interplay between population growth, social upscaling, ecosystem engineering and climate variability, the evoked demographic–resource-complexity mechanisms have not been empirically tested. By integrating proxy data for population sizes, palaeoclimate and internal societal factors into empirical modelling approaches from the population dynamic theory, we evaluated how endogenous (population sizes, warfare and social upscaling) and exogenous (climate) variables module the dynamic in past agrarian societies. We focused on the inland Atacama Desert, where populations developed agriculture activities by engineering arid and semi-arid landscapes during the last 2000 years. Our modelling approach indicates that these populations experienced a boom-and-bust dynamic over the last millennia, which was coupled to structure feedback between population sizes, hydroclimate, social upscaling, warfare and ecosystem engineering. Thus, the human–environment loop appears closely linked with cooperation, competition, limiting resources and the ability of problem-solving.

This article is part of the theme issue ‘Evolution and sustainability: gathering the strands for an Anthropocene synthesis’.

## 1. Introduction

Humans have become the dominant force on the functioning of the Earth system, altering biophysical processes till exceeding several planetary boundaries [1]. Although this state—the Anthropocene—is predominantly tied to the impact of contemporary industrialized societies [2], deep-time evolutionary evidence challenges this notion by emphasizing active/reactive human agencies since prehistoric times [3–6]. In this sense, the Anthroecology theory emphasizes coevolutionary processes, putting the socio-cultural niche construction at the core of the debate and, therefore, the pivotal role of long-term interplays between social upscaling and cooperative ecosystem engineering [7]. Simply put, as social systems scale up through time—in terms of population sizes, energy consumption, spatial aggregation, social hierarchy, group specialization and technological innovation—the capacity to access energy and materials from the ecosystems is constantly enhanced via environmental and cultural transformations enacted by cooperative social interactions [7,8].

Such positive feedbacks are related to population sizes through *per capita* resource share. As the population pressure increases, the intensification of ecosystem engineering is fuelled—by adjusting technologies or specialization—to enhance productivity, which ultimately increases demographic levels. The niche construction theory, however, predicts that even as ecosystem engineering enhances social wealth, the impact on the ecological inheritance could be either positive or negative. In fact, the trajectory of human populations is neither linear nor progressive in the long-run but punctuated by demographic boom-and-bust cycles, phases of growth/overshooting as well as of expansion/contraction in productivity [5,8–10].

All the above implies that the interplay between human population growth, social upscaling, and ecosystem engineering could be modulated by the interference of exogenous and endogenous factors. Archaeological and historical evidence attest for the role of climate variability in driving the rise and fall of past complex social systems (e.g. [11]); where rise (collapse) refers to sustained increase (loss) in population numbers [12]. Different pre-industrial societies indeed collapsed during periods of adverse climatic conditions, high population growth and resource depletion despite the deployment of technologies and social arrangements to enhance crop productivity [12–15]. The increased frequency of warfare is also documented as climatic fluctuations led to diminished resource availability [16–18]. Meanwhile, Turchin and co-workers [19–21] posit that demises and conflicts are part of rise–fall cycles inherent to complex societies brought about by offer–demand dynamics over strategic resources.

To our best knowledge, the nonlinear intervention of climate and warfare in the interplay between demography, social upscaling and ecosystem engineering has not been empirically tested. To make strong cases for such dynamics in past agrarian societies, it is critical to formally examine the impact of climate variability and inherent societal variables on the *per capita* supply of strategic resources, and how these effects propagated into the demography, complexity and social stability. Therefore, the climate-ecosystem-social-demography feedback could be explored using models based on population dynamic theory as conceptual devices.

Here, we evaluate the long-term interplay between endogenous variables (population sizes, warfare, ecosystem engineering and social upscaling) and the exogenous impact of climate conditions over the last 1200 years. We adopted theoretical and methodological approaches from Population Dynamic Theory (PDT). In practice, we fitted dynamic models in which the effect of climate, social upscaling, ecosystem engineering and warfare intensity are evaluated as a function of past population sizes, because these variables act in conjunction with demographic levels through antagonistic interactions (competition versus cooperation) [22].

**Figure 1.**
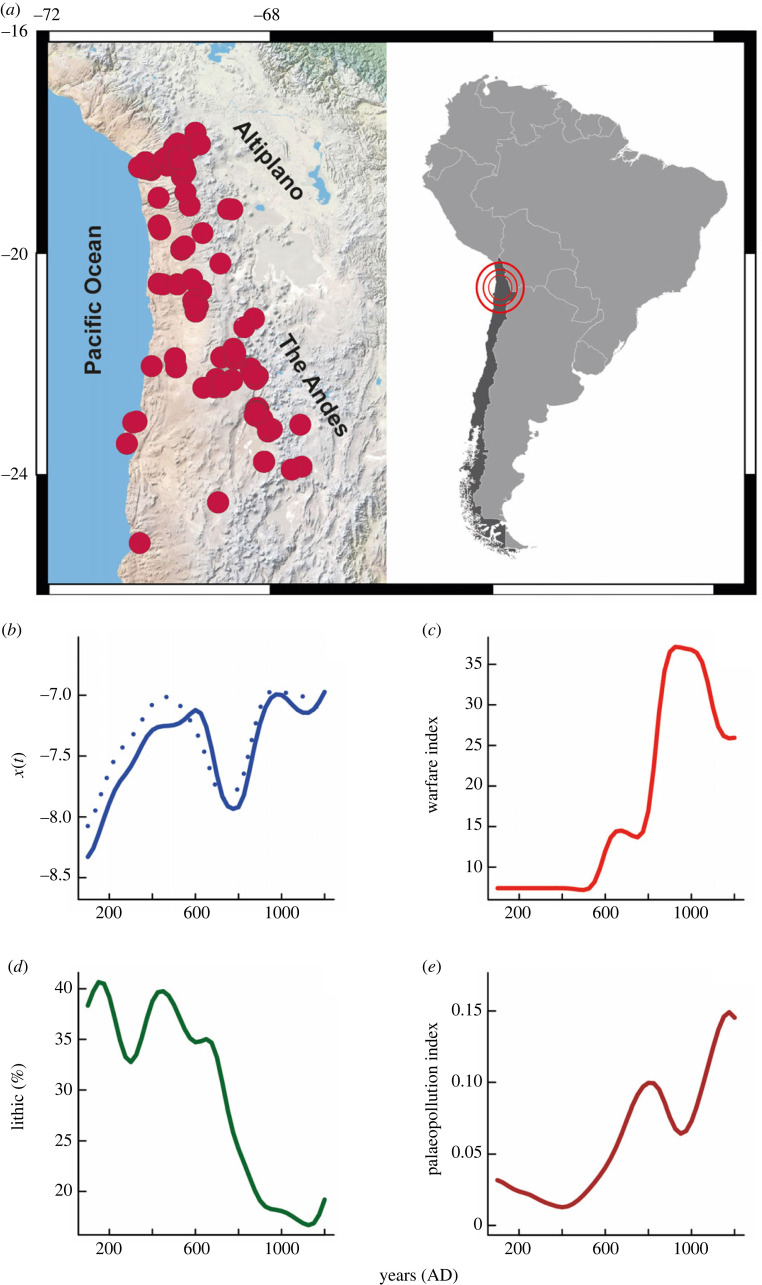
Study area and smoothed time series. (*a*) Spatial distribution of archaeological sites that provide radiocarbon dates used in our palaeodemographic reconstruction for the inland Atacama Desert. (*b*) Time series for past population levels inferred from the normalized (solid curve) and unnormalized (dotted curve) summed probability distributions (SPDs) of calibrated ^14^C-dates. (*c*) Time series for regional conflict incidence. The warfare index was calculated from bioanthropological data for interpersonal violence [23,24]. (*d*) Proxy for regional hydroclimate conditions based on lithic concentrations in the SO147–106KL marine core [25]. (*e*) Time series for the palaeopollution index as a proxy for social upscaling. This index builds on enrichment factors for lead and mercury from Illimani ice-cores [26,27].

We focused on the inland Atacama Desert (16°−25°S; figure 1*a*), where populations had developed agriculture activities since approximately 1500 BC by engineering a landscape of abrupt reliefs and diversified arid bioclimates—from extreme hyperaridity over the lowlands to cold semi-arid Andean highlands. These agrarian populations followed a trajectory marked by ever-increasing trends in demographic levels, social upscaling, ecosystem engineering, technological innovations and agriculture intensification [28]. In parallel, conflict increased, likely driven by ecological constraints on the availability of freshwater and arable lands [23,24], and the long-term population dynamic has been linked to cascade impacts from changes in hydroclimate on the ecosystem services [29]. Still, these cause–effect mechanisms and resource–demographic interactions evoked to explain the population and conflict dynamics in the Atacama Desert remain speculative as sustained on simplistic evidence for causality.

## 2. Methods

### (a) Past population sizes

We conducted a palaeodemographic reconstruction based on proxy data for population sizes. We specifically use the ‘ages-as-data’ approach, which assumes that the accumulation of archaeological radiocarbon dates on a given area is a function of the intensity of human activities on the landscape as well as of the energy consumption/production [30,31]. The summed probability distributions (SPDs) of archaeological ^14^C-dates have gained wide popularity over other existing sources to estimate demographic trends in prehistoric societies from different regions of the globe [14,28,32–35]. This is because SPDs provide statistically validated and chronologically constrained time series, yielding continuous sequences of high-resolution demographic data that allow us to compare and correlate dynamics in past socio-ecological systems.

To compute SPDs, we considered 418 radiocarbon dates on terrestrial samples (i.e. charcoal, plant remains, wood) from 199 archaeological sites along the inland Atacama Desert (figure 1). This set draws upon previously published databases [28,36] that have been recently indexed to the global P3K14C database [37] (available at https://github.com/people3k). We specifically used the unscrubbed subset contained within the P3K14C database (Dataset1, electronic supplementary material). The overall sampling intensity (0.35 dates 100 yr^−1^ 100 km^−2^) is significantly higher than those obtained in other palaeodemographic studies (0.13–0.22 dates 100 yr^−1^ 100 km^−2^; [38]). Such sampling intensity makes us confident that we can infer palaeodemographic trends that are representative for a vast region of the inland Atacama Desert over the last 1000 years.

We computed SPDs in the Rcarbon package for R [39]. ^14^C-dates were calibrated using the SHCAL20 calibration curve. To avoid any edge effect, we calibrated and summed dates over the time range between AD 100 and AD 1450. We applied a bin size of 50 years to reduce biases from overrepresentation of specific chronological phases and/or archaeological sites. A 100-year rolling mean was also applied to offset the contribution of calibration biases and the so-called sampling error [34]. Two time series of SPD were obtained to explore the potential impact of uncertainties brought about by changes in the slope of the SHCAL20 calibration curve, ultimately related to fluctuations in atmospheric radiocarbon production rather than genuine palaeodemographic patterns [39]. That is, we generated time series for SPD either for normalized or unnormalized calibrated ^14^C-dates (Dataset2, electronic supplementary material).

Because of the nature of ^14^C dates, the adoption of time-series approaches to infer palaeodemographic trajectories from SPDs is criticized by some research groups. Specifically, these claim that uncertainties brought about by calibration effects as well as measurement and sampling errors yield biased sources of information incompatible with statistical properties (autocorrelation and stationarity) required to implement such analyses [40–42]. However, these limitations could be overcome by offsetting uncertainties following standard procedures (as described above). Contemporary time series for human and other animal populations are usually non-stationary [43], which is routinely tackled by detrending. Low temporal autocorrelation is solved by evaluating the relative role of different factors on the observed variability pattern, and in turn analysing dynamics with different time-dependent variables. During the past years, several sophisticated statistical techniques have been proposed to test the impact of data uncertainties in palaeodemographic reconstructions based on SPDs, specifically ideated to test if ‘observed’ fluctuation pattern in the distribution of SPDs arises from random sampling error and calibration effects (e.g. [40,44–46]). Even when these represent valuable advances, the overcomplexities of such techniques reflect the wrong impression that archeological, chronological and/or palaeoecological data are uniquely and intrinsically subject to uncertainties.

The ecology research community studying the contemporary dynamics of natural populations also confront data uncertainties as counts for the number of individuals (as census) are rarely available. So, estimates of population sizes are usually based on incomplete and limited information. For instance, the progress of the PDT and its contributions to the biodiversity conservation has grounded on a diverse array of indirect proxies for populations sizes: biological remains (faeces, fur), biomass teledetection, aerial surveys, mark–release–recapture approach, sighting/game/fishery landings records, line transect, quadrant surveys or estimations of functional habitat. Hence, critiques based on the sampling error and its role in SPDs estimation is a transversal issue present in other disciplines. Still, it is important to note that any statistical analysis depends on assumptions about the data, but there is no analysis where everything is already known about the data such as distribution, likelihood functions or even the exact meaning of the dependent variables. The art of statistical analysis is to make reasonable assumptions given the structure of the data and then, *a* *posteriori*, evaluate the predictive power of a model. A wrong assumption is always a problem with any data in any system. A careful analysis of the raw data must be made before making conclusions about its utility, and most of these problems arise when researchers try to compare fitted parameters to data from different systems. The analysis of a single system, and specifically the rate of change of the focal variable, is the focus of this work. So, here we discuss the whole dynamics of the system and not specific values or parameters. In this sense, all alternative approaches to produce SPDs [40,44–46] involve more assumptions (and even more risky ones) than the simple approach applied here. At the end of the day, it is the predictive power of more effective metrics that evaluates the performance of a model and not the set of *a priori* assumptions of the researcher.

### (b) Hydroclimate data

Palaeoclimate reconstructions available for the Atacama and adjacent highlands show alternation of positive and negative hydroclimate anomalies at centennial timescales over the last 1300 years [47–50]. Many of these proxies, however, are temporally discontinuous, poorly resolved and inform qualitatively—and comparatively—the direction of past hydroclimate changes (i.e. wetter versus drier). All of this hinders the absolute magnitude/intensity for hydroclimate anomalies, making it difficult to integrate such palaeoclimate data into dynamical population models. For this reason, we adopted the premise that proxies for overall climate forcers describe large-scale conditions (e.g. ‘packages of weather’ *sensu* [51]). Observational data indicate that water availability, productivity and agricultural production over the western Andean slope is modulated by the El Niño Southern Oscillation (ENSO) at interannual scales [52–55]. Thus, reconstructions for past changes in ENSO activity represent feasible substitute records for the long-term hydroclimate variability over the inland Atacama Desert. We selected the annually resolved concentration of terrestrial lithics accumulated in marine sediments (core SO147–106KL) approximately 80 km offshore of Peru at approximately 12°S [25] (see Dataset3, electronic supplementary material). The accumulation of terrestrial clast in sediments of the Peruvian shelf results from riverine sedimentary inputs, so its variability through time accounts for changes in the intensity and magnitude of coastal rainfall. Therefore, decreased (increased) accumulations of terrestrial clasts reflect reduced (increased) sediment transport due to decreased (increased) precipitation along the Peruvian coast, which is brought about by La Niña-like (El Niño-like) conditions in the Equatorial Eastern Pacific [25]. The relationship between clast concentrations (lithics %) in the SO147–106KL core, hydroclimatic conditions and ENSO activity is, however, inverse over the inland Atacama Desert. That is, reduced (increased) lithic percentages, and in turn prevailing La Niña-like conditions, argue for positive (negative) hydroclimate anomalies across the region between 16° and 25°S (see [29] for further explanations).

### (c) Social upscaling

A time series for the palaeopollution index was produced to grasp quantitatively the long-term trend in social upscaling over the inland Atacama (Dataset4, electronic supplementary material). In this region, past pollution by heavy metals is expected as the result of the interplay between the intensity of metallurgy activities, social upscaling and cooperative ecosystem engineering [28]. Actually, the regional prosperity for the metallurgy involved the procurement of local/imported ores processed with sophisticated devices (wind-sourced furnaces) and techniques (e.g. smelting, cupellation) in delimited production areas by highly specialized labour, all of which is ultimately a function of food surplus [28]. Just like the rest of the Andean Cultural Area, past pyrometallurgy in the inland Atacama stimulated complexity by providing goods for ritual, ornamental and social differentiation purposes, but to a much lesser extent toolkits for enhancing food production and access to natural resources [56,57]. This sharply contrasts with productivity pursuits of the Old World prehistoric metallurgy as well as with the commercial metallurgy regime introduced in the region since colonial times [28,57].

Our palaeopollution dataset aggregates sequential (at 50-year intervals) estimates for crustal-normalized concentrations (i.e. Enrichment Factors) for lead (Pb) and mercury (Hg) reconstructed from two geochemical characterizations of Illimani ice-cores (approx. 16°S [26,27]). By this means, the dataset brings together data that account only for the contribution of traditional pyrometallurgical activities in the deposition of heavy metals emitted during the production of native copper alloys and silver. To obtain a time series for a palaeopollution index that describes the trajectory for social upscaling and cooperative ecosystem engineering, enrichment factors estimated for Hg and Pb in both geochemical reconstructions were min–max normalized, and then merged by averaging normalized values [28].

### (d) Conflict data

The warfare-intensity time series builds on data for interpersonal violence inferred from the bioanthropological record from San Pedro de Atacama oases [23] and the Azapa valley [24,58] through the period AD 100–1400 (Dataset5, electronic supplementary material). In practice, our dataset concatenates scores for the frequency of cranial and postcranial traumas, which represent archaeological proxies for physical conflict during times of social tensions (e.g. [24,59]). Although this set is the most comprehensive source of data on interpersonal violence for the inland Atacama (*n* = 596 records), we recognize that it might be subject to biases in spatial and temporal coverages. Such limitation implies that patterns reconstructed here could account for a fraction of the warfare dynamic over the inland Atacama, and, in turn, these should be taken at face value for available data at the moment.

We accepted the quality and reliability of trauma data reported in the original sources as well as their chronologies. Since the temporal distribution of frequency for injuries are given in chrono-cultural periods defining the regional cultural sequence (Late Formative–Late Intermediate Period), we transformed such broad and large time-step data into an annual scale through linear interpolation. Then, we generated a time series that draws upon a conflict index calculated by averaging min–max normalized frequencies of injuries available for the San Pedro de Atacama oases [23] and Azapa valley [24,58].

### (e) Data treatment

Our population models should be fed with data that capture major trends in population levels as well as the long-term trends in mean hydroclimate, social complexity, cooperative ecosystem engineering and conflict incidence. This implies that the high-resolution noise source of variability in each time series should be removed before implementing our population dynamic models. That is, the normalized and unnormalized SPD time series were sectioned into time-step intervals of 25 years [14]. Such fixed time-step between observations (i.e. time delay parameter) corresponds to one generation time (approx. 25 years), which is relevant for portraying dynamic processes in human populations by offsetting redundancy and irrelevance in demographic time series, to significantly reduce the problem of artificially inflating the *p*-values and of using calendar years as sample size.

To link hydroclimate (lithic %), warfare incidence (conflict index) as well as social complexity and ecosystem engineering (palaeopollution index), all times-series were smoothed by fitting a cubic spline function [60]. Spar times used to fit cubic spline functions, however, varied according to the temporal resolution of each dataset. The annually resolved time series of lithic percentages (lithic %) was smoothed by setting an intermediate spar parameter of 0.65; lower and larger spar parameters were used for smoothing palaeopollution (0.45) and warfare (0.70) time series, respectively. Datasets for lithic and conflict percentages were then resampled at 25-year intervals.

### (f) Population dynamic models

We begin with a simple, non-structured theoretical model describing the effects of intra-specific competition on the dynamics of a population growing in a finite environment [61,62] that is a time-discrete version of the original continuous-time model of Verhulst [63]:

2.1 $$x_{t+1}=x_{t}\cdot r_{m}\cdot e^{\left[-s\cdot (1-k)\cdot x_{t}\right]}$$

where *x_t +_*_1_ is the population size at time *t* + 1, *r_m_* is the (mean) potential reproductive rates of the individuals (i.e. when no competitors exist), *s* is a positive constant representing the individual resource requirements, and *k* is a positive constant 0 < *k* < 1 that reduces the potential reproductive rates each time an additional individual is added to the population, the lower the *k*-value the faster is the decrease in reproductive rates [61,62]. In fact, the expression *s*(1 − *k*) in equation (2.1) is a measure of the intensity of competition among the individuals of the population [61] and can be written as a simple constant *c* [62]. Because the net rate of change from generation *t* to *t* + 1 is measured as the ratio *x_t_*_+1_/*x_t_* = *r_t_*, the model of equation (2.1) can be written as,

2.2 $$r_{t}=r_{m}\cdot e^{\left[-c\cdot x_{t}\right]}$$

The model describing the effects of intra-population cooperation can be represented as the opposite force of equation (2.2); using the same ecological base the discrete time-step model of cooperation is

2.3 $$x_{t+1}=x_{t}\cdot r_{m}\cdot e^{\left[-z\cdot (1-k^{\prime })\cdot 1/x_{t}\right]}$$

where *r_m_* is the mean net reproduction rate, and *z* is a positive constant that represents the effect that some environmental hazards impose on the individuals of the population: for example, the defences or threats of some important resources for the individuals. Therefore, in this case, and in opposition to equation (2.1), *k′* is a positive constant 0 < *k′* < 1 that increases the potential reproductive rates toward the maximum reproductive rate *r_m_*, each time an additional individual is added to the population. The lower the *k′* value the faster is the increase in reproductive rates. In fact, both the expressions, *s*(1 − *k*) in equation (2.1) and *z*(1 − *k*′) in equation (2.2), can be written as simple constants *c* and *w*, being the intensity of competition and the amount of cooperation needed to overcome the environmental hazards, respectively. The higher the *c* value the faster is the decrease in population growth rates with population density. The higher the *w* value the higher the population size needed to increase population growth rates. Because the net rate of change from generation *t* to *t* + 1 is measured as the ratio *x_t_*_+ 1_/*x_t_ = r_t_*, equations (2.1) and (2.3) can be combined in a single model including the effects of both terms, competition and cooperation. Again, *r_m_* is the mean net reproduction rate, whereas *w* is a measure of the intensity of cooperation among individuals of a population (the opposite force to *c* in equation (2.2)). Equations (2.2) and (2.3) can be thus combined in a single model including the effects of both terms, competition and cooperation, as

2.4 $$r_{t}=r_{m}\cdot e^{\left[-c\cdot x_{t}-w\cdot 1/x_{t}\right]}$$

For analytical convenience, we write equation (2.4) in terms of the logarithmic (*per capita*) reproductive rate log_e_ (*r_t_*) = *R_t_* and log_e_ (*x_t_*) = *X_t_* as:

2.5 $$R_{t}=R_{m}-c\cdot e^{\left(X_{t}\right)}-w\cdot e^{\left(-X_{t}\right)}$$

The shape of the reproductive curve *R-X* is determined only in terms of these three parameters *c, w* and *R_m_—*which in this equation is the intercept of the curve on the *R-X* phase plot*.* Actually, some of the model parameters have a clear socio-ecological and population dynamic interpretation such as the logarithmic (mean) maximum reproductive rate (*R_m_*), the intensity of the intra-population competition (*c*) and the intensity of demographic cooperation (*w*). The model of equation (2.5) can be modified to introduce the effects of changes in hydroclimate (lithic % as a proxy for water availability), warfare intensity (conflict index) and social complexity/cooperative ecosystem engineering (palaeopollution index) on the parameters *c* and *w*, by assuming a simple linear function effect of each factor on these two parameters.

2.6 $$R_{t}=R_{m}-(c+\alpha \cdot z_{t})\cdot e^{\left(X_{t}\right)}-(w+\beta \cdot z_{t})\cdot e^{\left(-X_{t}\right)}$$

where *z_t_* represents each of the three exogenous factors (*z_t_* = lithic % as proxy of climate, *z_t_* = warfare and *z_t_* = social upscaling). The parameter *α* can be interpreted as how a given environmental factor (i.e. hydroclimate, warfare and social upscaling) modifies the individual resource requirements (*s*) or how an additional individual reduces the *per capita* growth rates (*k*). Therefore, positive (negative) *α* values increased (decreased) the intensity of intra-population competition with changes in the hydroclimate (droughts), warfare and social upscaling (palaeopollution index). In the same vein, parameter *β* can be interpreted as how some exogenous factors (i.e. hydroclimate, warfare and social upscaling) modify the *per capita* effect of some environmental hazard and the manner that individuals cooperate (*k*′). Hence, positive (negative) *β* values indicate increases (reductions) in the level of cooperation needed to overcome potential environmental hazards, such as droughts and social conflicts.

We fitted the log-transformed SPD time series to equation (2.6) with nonlinear regressions using the *nls* (nonlinear least squares) library in the R programming language [64]. Models were ranked according to the second-order Akaike's information criterion (AICc; see [65] for details), and we calculated Akaike's weights (*w_i_*) to infer the relative likelihood of each model [65]. Finally, by using a multimodel inference approach [65,66], we estimated the relative importance of each predictor across all candidate models and identified the variable(s) that might be driving the system dynamics. This enables quantifying the probability that a given hypothesis is explaining the observed dynamics. It is important to notice that in the case of nonlinear models, the *R^2^* calculated for each model cannot be used to determine goodness of fit or model performance [67], and consequently we focused primarily on the AIC_c_ and *w_i_* results (see below).

### (g) Model validation

We compare and validate the models by simulating the total trajectory predictions initiated with the first observed value of the time series and running the algorithm using each model with their estimated parameters to obtain the time series' remaining simulated values. This implies that we are able to evaluate how good is a model in predicting the total trajectory of the population dynamic for the whole 1000 years period by not using the raw or smoothed SPD time series, but only the first SPD value in the corresponding time series along with environmental variables in *t* or *t*− accordingly.

In all simulations, the accuracy of predictions was assessed using coefficient of prediction *σ*^2^ [68]:

2.7 $$\sigma^{2}=1-\frac{\sum_{i=1}^{n}{\left(O_{i}^{*}-O_{i}\right)}^{2}}{\sum_{i=1}^{n}{\left(\bar{O}-O_{i}\right)}^{2}}$$

*O_i_* indicates observed data from the testing dataset, $O_{i}^{*}$ denotes the model predictions, $\bar{O}$ is the mean of the observations, and *n* is the number of data to be predicted. Coefficient of prediction *σ*^2^ is 1 when predicted data are equal to the observed data, 0 when the regression model predicts and the data average, and negative if the predictions of the model are worse than the data mean (scripts and data fully available).

Best models were selected considering the Akaike Information Criterion corrected for the small sample size (AICc) and the coefficient of prediction (*σ*^2^) of the simulated total trajectory predictions initiated with the first observed value of the time series. The parameter $\Delta {\mathrm{AIC}}_{c}$ is calculated as the difference between the model's AICc and the lowest AICc.

## 3. Results

Normalized and unnormalized SPDs for calibrated ^14^C-dates reproduce practically identical patterns, thus both revealing a convergent boom-and-bust dynamic in the inland Atacama Desert over the period AD 100–1200 (figure 1*b*). Specifically, we evince successive phases of growth and collapse that lasted between AD 400 and AD 600 years. From AD 100, populations underwent a steady demographic expansion, peaking at around AD 500–600. From around AD 600 and AD 800, population sizes decreased rapidly, leading to the first demographic stagnation–collapse phase. Then, the accelerated recovery in population numbers between AD 800 and AD 1050 marks a second demographic expansion and a posterior stagnation phase (figure 1*b*).

By exploring and comparing trends via simple visualization, the relationship between population dynamics, hydroclimate, conflict incidence and social upscaling is not straightforward. The population boom at AD 100–600 indeed coincides with an interlude of lowest warfare intensity (AD 100–500; figure 1*c*) and decreased water budgets over the inland Atacama (figure 1*d*). Actually, high clast concentrations in the SO147–106KL core (greater than 33%, figure 1*d*) attest to persistent El Niño-like conditions (AD 100–700) over the duration of the first expansion phase; whereas, during the stagnation–collapse phase at AD 600–800, as the intensity of conflict increased rapidly (figure 1*c*), population levels dropped (figure 1*b*) even though more favourable hydroclimate conditions might have persisted as the Pacific began to transition into a La Niña-like mean state (AD 700–900; figure 1*d*). By contrast, the second demographic expansion (AD 800–1050) takes place over a period in which wetter conditions prevailed (figure 1*b*), and the conflict intensity keeps increasing, peaking at AD 850–1050 (figure 1*c*).

As is for the warfare intensity (figure 1*c*), the social upscaling/ecosystem engineering shows an overall uptrend over the last 1200 years (figure 1*e*). Still, mid-term variation patterns are evident. Low values for the palaeopollution index occurred at the beginning of the first demographic expansion (figure 1*b*), but the rapid increase from AD 400 to AD 850 suggests that social upscaling/ecosystem engineering accelerated as the population grew and El Niño-like conditions persisted along the Eastern Tropical Pacific (figure 1*d*). This pattern, however, extended even over the first demographic stagnation–collapse phase (AD 600–800), when the palaeopollution index reached the maximum values (0.9–0.11; figure 1*e*) recorded for the period AD 100–850. Then, the drop in the palaeopollution index at AD 850–1000 (figure 1*f*) suggests a short-lived contraction in social upscaling that matches with the second demographic expansion. This trend then reversed, as indicated by the accelerated increase in the palaeopollution index since AD 950, which peaked between AD 1100 and AD 1200 (figure 1*e*). Interestingly, from AD 850 onwards social upscaling covaried inversely with population growth, conflict intensity and hydroclimate conditions.

**Figure 2.**
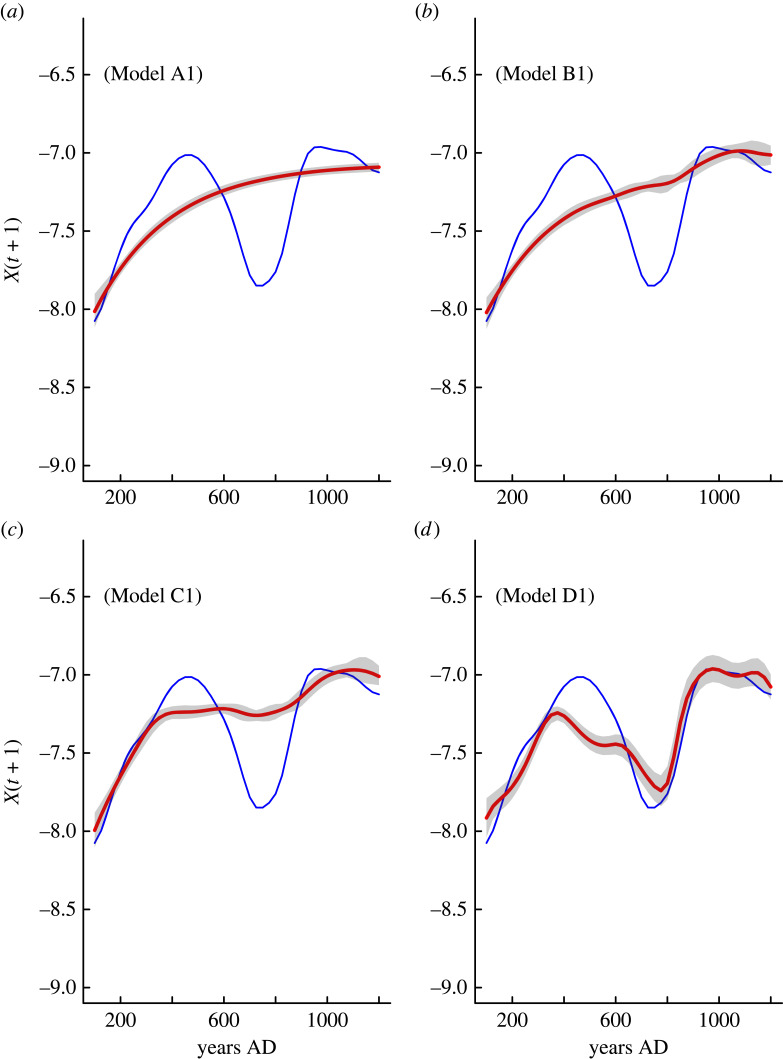
Comparison of observed unnormalized SPD data (blue curves) and predicted (red curves) population dynamic derived from the A_1_ model (table 1) that includes a pure endogenous dynamic (*a*); the effect of warfare and hydroclimate on parameter *c* (Model B_1_; *b*); and the effect of warfare and hydroclimate on parameter *c* and social upscaling on parameter *w* (Model C_1_; *c*). Panel *d* incorporates the interplay of warfare and hydroclimate on parameter *c,* and social upscaling and warfare on parameter *w* (Model D_1_).

**Figure 3.**
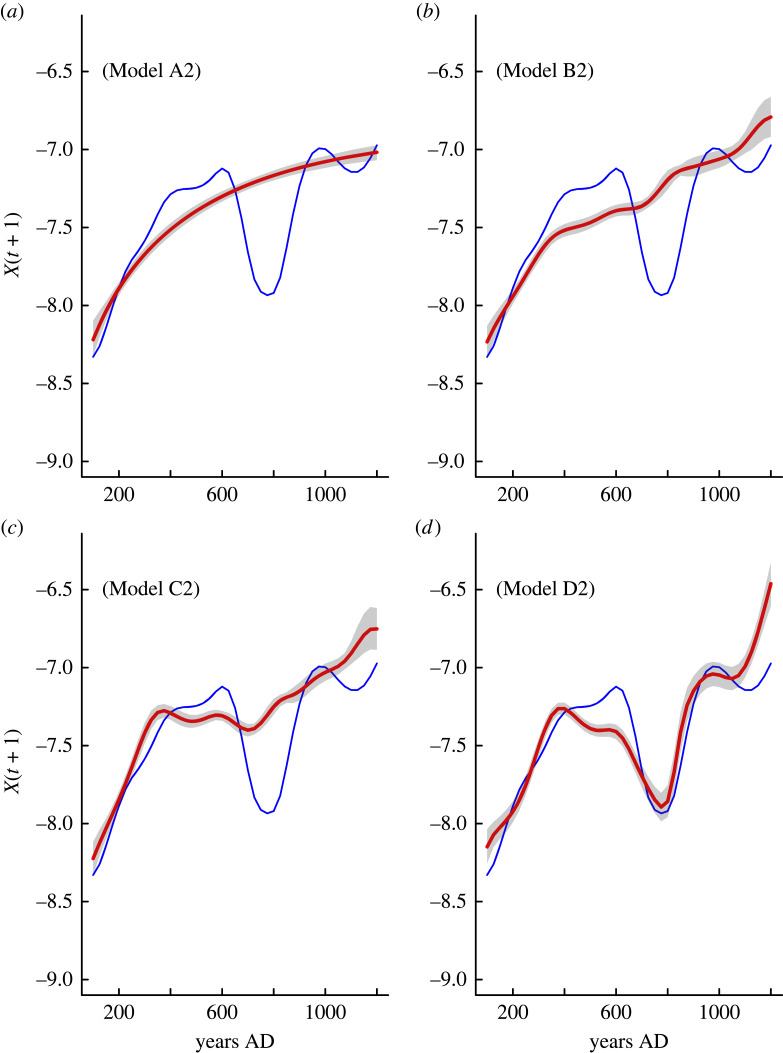
Comparison of observed normalized SPD data (blue curve) and predicted (red curves) population dynamic derived from the A_2_ model (table 1) that includes a pure endogenous dynamic (*a*); the effect of warfare and hydroclimate on parameter *c* (Model B_2_; *b*); and the effect of warfare and hydroclimate on parameter *c* and social upscaling on parameter *w* (Model C_2_; *c*). Panel *d* incorporates the interplay of warfare and hydroclimate on parameter *c*, and social upscaling and warfare on parameter *w* (Model D_2_).

Population dynamic models including the effect of warfare and hydroclimate on the intra-population competition strength perform poorly (table 1 and figures 2*a–c*, 3*a–c*). The same is true for the effect of social upscaling on demographic cooperation. The best-fitted model (lowest AICc and highest *w_i_*) includes the positive effect of droughts and warfare intensity on the intra-population competition strength (parameter *c* in equations (2.1)–(2.6)) as well as the effects of social upscaling and conflict on the demographic cooperation (parameter *w* in equations (2.1)–(2.6)). The term containing *c* plus the hydroclimate and warfare tends to increase with high lithic percentages (indicative of drought) and warfare frequency, indicating that both factors can increase the intensity of intra-population competition, while the term containing the coefficient *w* is positively affected by social upscaling and negatively by warfare intensity. In turn, ecosystem engineering/social upscaling increases the level of cooperation needed to overcome an exogenous hazard or threat, while conflict affects it negatively.

**Table 1.** Population models (equations (2.5) and (2.6)) fitted to SPD time series from the inland Atacama Desert. Models A: include a pure endogenous dynamic ($R_{t}=R_{m}-c\cdot e^{\left(X_{t}\right)}-w\cdot e^{\left(-X_{t}\right)}$). Models B: the effect of warfare and hydroclimate on the parameter *c* ($R_{t}=R_{m}-(c+\alpha_{0}\cdot z_{t}+\alpha_{1}\cdot {z^{\prime }}_{t})\cdot e^{\left(X_{t}\right)}-w\cdot e^{\left(-X_{t}\right)}$). Models C: the effect of warfare and hydroclimate on parameter *c* and social upscaling on parameter *w* ($R_{t}=R_{m}-(c+\alpha_{0}\cdot z_{t}+\alpha_{1}\cdot {z^{\prime }}_{t})\cdot e^{\left(X_{t}\right)}-(w+\beta_{0}\cdot z^{\prime }{{\;}^{\prime }}_{t})\;\cdot \;e^{\left(-X_{t}\right)})$. Models D: the interplay of warfare and hydroclimate on parameter *c* and social upscaling and warfare on parameter *w* ($R_{t}=R_{m}-(c+\alpha_{0}\cdot z_{t}+\alpha_{1}\cdot {z^{\prime }}_{t})\cdot e^{\left(X_{t}\right)}-(w+\beta_{0}\cdot z^{\prime }{{\;}^{\prime }}_{t})+\beta_{1}\cdot {z^{\prime }}_{t})\cdot e^{\left(-X_{t}\right)}$). Subscripts 1 and 2 indicate models adjusted using unnormalized and normalized SPD time series, respectively. Parameter values are given in the columns of the table. The model notations are: *R*_m_, the logarithmic (mean) maximum reproductive rate; *c*, the intensity of intra-population competition; *w*, the intensity of population cooperation; *α*_0_ and *α*_1_, the effects of hydroclimate and warfare on *c;* *β*_0_ and *β*_1_, the effects of social upscaling and warfare on *w*, respectively. AICc, Akaike Information Criteria corrected for the small sample size; *w_i_*, Akaike's weights; *σ*^2^, the coefficient of prediction predictions initiated with the first observed value of the time series. Models with the highest support are highlighted in italics.

model	*R_m_*	*C*	*W*	*α*_0_	*α*_1_	*β*_0_	*Β*_1_	AICc	*Δ*AIC	*w_i_*	*σ*^2^
A_1_	0.028	66.97	−0.00003					−96.06	13.55	0	0.2
B_1_	0.15	216.1	0.000003	−0.22	−1.87			−92.34	17.27	0	0.3
C_1_	0.20	14.74	−0.000013	6.70	0.95	0.0005		−92.43	17.18	0	0.5
*D_1_*	*−0.81*	*−1941*	*−0.0002*	*29.9*	*35.0*	*0.0024*	*−0.00002*	*−109.61*	*0.0*	*1*	*0.8*
A_2_	−0.02	88.16	−0.000034					−85.64	38.70	0	0.4
B_2_	0.096	−224	−0.00001	9.60	4.48			−83.65	39.69	0	0.4
C_2_	0.28	−437	−0.00002	20.3	8.38	0.0065		−86.96	36.38	0	0.6
*D_2_*	*−0.75*	*−2625*	*−0.00014*	*43.3*	*48.0*	*0.0024*	*−0.00002*	*−123.34*	*0.00*	*1*	*0. 8*

The best-fitted population dynamic model attests for the interaction between endogenous and exogenous variables in driving the population dynamic in the inland Atacama Desert during the last 1200 years (figure 2*d*). Two major predictions arise from this model. First, that negative hydroclimate anomalies and increased conflict intensity reduce the long-term food production (crop yields and/or availability of arable lands). Second, that ecosystem engineering/social upscaling leads to demographic cooperation, which is counteracted by increases in the warfare intensity. Such a mechanism is supported by the cumulative *w_i_* and the coefficient of prediction $\left(\sigma^{2}\right)$ obtained for this full model (table 1) that displays the highest probability (100%) of being included as the best model in comparison to the low statistical support for the rest of other competition–cooperation models (table 1). Actually, total trajectory predictions indicate that it is capable of capturing the particular demographic pattern of expansion–collapse that agrarian populations maintained for almost a millennium over the inland Atacama Desert (figures 2, 3 and table 1).

## 4. Discussion and conclusion

By the explicit integration of proxy data for population sizes, climate and internal societal variables with the empirical modelling approaches from the PDT, here we provide new insights into the feedback relationships in the dynamic of past agrarian societies. In particular, we verify that the population dynamic for the inland Atacama Desert was coupled to the interplay between hydroclimate, social conflict and social upscaling/ecosystem engineering.

Our modelling approach suggests that competition intensity is stronger during droughts and times of warfare, while cooperation is enhanced during periods of peace and high social upscaling. Hence, neither the effect of climate, ecosystem engineering nor warfare intensity are sufficient by their own to explain the expansion–stagnation–collapse dynamic observed for the inland Atacama. By contrast, our results agree with the notion that the human–environment loop is closely linked with cooperation, competition, limiting resources, environmental hazards and the ability of problem-solving [69]. Several authors indeed argue for structured feedback between both dynamics, stressing the correspondence between events of population growth, technological innovations and land-use intensification [5,8–10,70–72]. So, the social upscaling in the inland Atacama emerges from the interplay between large population sizes, ecosystem engineering, climate change and the adoption of technological innovations.

In this sense, our results support theoretical models proposed by Marquet *et al*. [73] and Santoro *et al*. [29] to explain the emergence of cultural complexity and adaptative strategies in the region during the past 14 000 years. We effectively evince that technology, demography and ideologies—that together articulate adaptative strategies—interacted with ecosystem services, and in turn with hydroclimate, apparently leading to socio-cultural continuities/discontinuities. This study, however, contributes additional elements to understand the relationship between hydroclimate, population sizes and social internal factors. The expansion–stagnation–collapse appears as a novel feature not previously noticed in early palaeodemography reconstructions aimed at contrasting overall patterns between hunter-gatherers versus agricultural periods [36,74]. Because such palaeodemography time series feed into adaptative and complexity models, the link between population sizes, hydroclimate and technology in these conceptualizations is, therefore, masked. Marquet *et al*. [73] and Santoro *et al*. [29] emphasize the positive feedback of hydroclimate on the population growth, which effect is propagated to the demography, technological innovations and complexity. Although water availability represents a limiting factor for crop productivity in extreme arid environments, we attest for a marginal effect of positive hydroclimate anomalies on the population growth during the expansion phases.

Opposite hydroclimate conditions prevailed during demographic expansions recorded at AD 200–600 and AD 800–1050 (figure 1*d*). These trends are replicated by independent palaeoclimate reconstructions, showing variable hydrological conditions at AD 200–600, and then a widespread positive hydroclimate anomaly that matches with the chronology for the Medieval Climate Anomaly [47,75–77]. The common denominator in both expansions is, however, that the accelerated population growth occurs in synchrony with new technologies and cooperative forms that were added to the production system. This implies that food production, population growth, innovations and cooperative behaviours are linked in such a manner that the capital for landscape engineering accumulated and increased.

By 500 BC, the production system in the inland Atacama already included worked crop fields artificially irrigated by complex irrigation networks to cultivate exotic Mesoamerican and Andean cultigens, small-scale husbandry of domesticated camelids, pottery and metallurgy industries, trans-Andean exchange networks, but also population aggregations in incipient sedentary settlements with architecture [28,78]. Shortly before the first expansion phase all these practices escalated. Still, the production system was further enhanced by the introduction of furrowed cultivation fields, wind-sourced smelting furnaces and the mesquite-tree agroforestry (*Neltuma* spp.) to fertilize and prevent soil degradation/desiccation [28,79]. The second expansion occured after the hydraulic technology improved (dams, perched channels), terraced agriculture emerged, *Neltuma* agroforestry intensified, metallurgy diversified, new edible cultigens and regional maize varieties were introduced, and the crop yield was enhanced through natural fertilizers [28,78,80,81]. This process was accompanied by the emergence of local entities (e.g. Arica culture, Pica–Tarapacá complex) that constituted consolidated expressions of the regional developments characterized by socio-political, territorial and material identities [82,83].

The demographic population model implemented here suggests that negative hydroclimate anomalies limit crop production—through water limitation—even when technological and ecosystems engineering capacities were available. For instance, the brief interruption of irrigated agriculture across the Atacama lowlands and the relocation of settlements to areas where water resources were more predictable coincides with a centennial-scale dry pulse dated at AD 650–850 [29,47,77]. The negative effect of hydroclimate is indirect but modulated by the ratio between population sizes and food production. This means that once the system approached the higher land productivity set by existing agriculture practices, the population growth stagnated and declined due to the reciprocal relationship between population pressure, *per capita* share of resources and competition strength.

We are unable to test directly if the warfare dynamic was driven by adverse hydroclimate conditions. Interpersonal violence is a chronic and endemic feature that varied spatially and temporally in intensity across the Atacama Desert [24,84,85]. Conflict incidence, however, escalated as population densities increased as well as sedentarism and agriculture intensified across an intrinsically constrained environment [23,24]. Even when conflict-driven risks were mitigated by developing defensive architecture, increasing exchange networks and/or relocating settlements and productive areas, the violence incidence was exacerbated when environmental conditions became unpredictable or adverse to sustain local production [23,24,82,86]. Our results are partially consistent with these appreciations. We attest for a relationship between population sizes, hydroclimate and warfare intensity, but the drier conditions act as an exogenous factor influencing the *per capita* share of resources.

Social upscaling appears as a key element underpinning the socio-cultural and demographic trajectories in the inland Atacama. For instance, population stability and adaptative continuities are evident in the archaeological record regardless of the stagnation–collapse phase [28,29]. The correlation between social upscaling and population stability is an expected result. The cross-cultural study by Freeman *et al*. [12] reveals that higher complexity levels in past agrarian societies, and in turn more investment in ecosystem engineering through technology intensification, leads to less vulnerability to external/internal perturbations and less severe demographic collapses. But, as the magnitude of the collapse increases, these societies are prone to large social transformations following the collapse event. In the case of the inland Atacama, the transformation process mostly involved the intensification of interregional exchange networks, adjustment of consumption practices (e.g. dietary items) and the relocation of settlements and production areas [29,82,83,87,88].

This study shows that the integration of proxy data for past demographic levels, hydroclimate conditions and societal variables into a statistical perspective, provides the means to test hypotheses on the resilience or collapse in societies that developed problem-solving capacities. Our analysis finds that the human–environment loop for the past agrarian populations from the inland Atacama Desert over the past 1200 years involved processes affected by population growth, warfare and hydroclimate as well as social complexity. This means that when dealing with projected scenarios for shared socio-economic trajectories this positive feedback should be explicitly considered—particularly the climate-related risks on economy and socio-political stability under a context of unprecedented human population growth accompanied by accelerated transformations in the functioning of the Earth system [89–92].

## Data Availability

The data are provided in electronic supplementary material [93].
